# A Digital Sensor Simulator of the Pushbroom Offner Hyperspectral Imaging Spectrometer

**DOI:** 10.3390/s141223822

**Published:** 2014-12-11

**Authors:** Dongxing Tao, Guorui Jia, Yan Yuan, Huijie Zhao

**Affiliations:** Key Laboratory of Precision Opto-Mechatronics Technology, Ministry of Education, School of Instrument Science & Opto-Electronics Engineering, Beihang University, Beijing 100191, China; E-Mails: buaaeye@aspe.buaa.edu.cn (D.T.); jiaguorui@buaa.edu.cn (G.J.); yuanyan@buaa.edu.cn (Y.Y.)

**Keywords:** hyperspectral, sensor simulator, Offner

## Abstract

Sensor simulators can be used in forecasting the imaging quality of a new hyperspectral imaging spectrometer, and generating simulated data for the development and validation of the data processing algorithms. This paper presents a novel digital sensor simulator for the pushbroom Offner hyperspectral imaging spectrometer, which is widely used in the hyperspectral remote sensing. Based on the imaging process, the sensor simulator consists of a spatial response module, a spectral response module, and a radiometric response module. In order to enhance the simulation accuracy, spatial interpolation-resampling, which is implemented before the spatial degradation, is developed to compromise the direction error and the extra aliasing effect. Instead of using the spectral response function (SRF), the dispersive imaging characteristics of the Offner convex grating optical system is accurately modeled by its configuration parameters. The non-uniformity characteristics, such as keystone and smile effects, are simulated in the corresponding modules. In this work, the spatial, spectral and radiometric calibration processes are simulated to provide the parameters of modulation transfer function (MTF), SRF and radiometric calibration parameters of the sensor simulator. Some uncertainty factors (the stability, band width of the monochromator for the spectral calibration, and the integrating sphere uncertainty for the radiometric calibration) are considered in the simulation of the calibration process. With the calibration parameters, several experiments were designed to validate the spatial, spectral and radiometric response of the sensor simulator, respectively. The experiment results indicate that the sensor simulator is valid.

## 1. Introduction

Compared with the whiskbroom imaging spectrometer, the pushbroom imaging spectrometer, with a higher signal-to-noise ratio (SNR), has been widely used in airborne and spaceborne remote sensing [1,2]. For the launched and developing pushbroom hyperspectral imaging spectrometers, the Offner dispersive imaging system is popular for its many advantages, such as low aberrations, a wide flat field, a compact size, and a high speed [3–7]. An Offner dispersive imaging system, which consists of three spherical and concentric surfaces: two concave mirrors and a classical convex grating, relies on the Offner unity magnification reflective relay [8].

Sensor simulators, translating the spatially and spectrally oversampled at-sensor radiance scene into the proposed digital number (DN) images, play an important role in forecasting the performance of the instrument when designing a hyperspectral imaging spectrometer [9]. A sensor simulator is generally composed of the simulation of imaging spectrometer's spatial response, spectral response and radiometric response. These responses are the key factors of the spectral quality metrics reported by Kerekes [10]. With responsibility for indicating the dispersive imaging ability of the hyperspectral imaging spectrometer, several sensor simulators were developed as part of the core sub-modules of the end-to-end remote sensing simulation model [11–13]. However, some problems still need to be resolved more, especially, in the simulation of the spatial response and the spectral response.

Generally, in the spatial response simulation, the operations of image positioning, spatial degradation and spatial resampling are considered [14]. Image positioning is a process of establishing the relationship between each detector element and the imaging projections on the ground surface in the imaging time. Spatial degradation, which is caused by the platform motion, optical system aberrations, slit size, detector element size, and so on, blurs the spatial image. Spatial resampling is a transformation process which outputs the resampled image with the ground sampling distance (GSD) of the proposed sensor. In most sensor simulators, point spread function (PSF), the matrix multiplication of the line spread functions (LSFs) in along-track and in across-track [15], is used to convolve with the oversampled image to implement the spatial degradation first. Based on the image positioning, the degraded image is then resampled to each imaging pixel's position with the GSD of the proposed sensor. However, the along-track and across-track directions of the sensor imaging data (also the along-track and across-track directions of the PSF) are different from the line and sample directions of the over-sampled input image. This difference will result in some negative effects (called direction errors here) on the simulated data. Therefore, using the existing spatial response simulation method, the degree of spatial blur of the simulated image will have some deviations from the proposed sensor. Although the direction error could be avoided by implementing the spatial degradation after the spatial resampling operation, according to the sampling theory, a serious extra aliasing effect caused by the undersampling will arise in that case [16].

Besides the spatial response simulation, the existing spectral response simulation also needs further development. Based on the assumption that a hyperspectral imaging spectrometer is linear shift invariant (LSI), the spectral response of the sensor is traditionally simulated by the convolution of the given SRF and the oversampled input spectra [17]. However, the SRF of the sensor is unknown in the sensor design state, only the assumed SRF can be used. In this case, the traditional sensor simulator cannot accurately forecast the imaging ability of the proposed sensor in the spectral dimension. Compared with the simulators of the multispectral imaging spectrometer and the panchromatic imager, the accuracy of the spectral response simulation is more important for the hyperspectral imaging spectrometer simulator [18]. Hence, the traditional spectral response simulation will limit the application potential of the sensor simulator.

In this work, a novel digital sensor simulator, which consists of a spatial module, a spectral module and a radiometric module, is presented for the pushbroom Offner hyperspectral imaging spectrometer. In order to balance the direction error and the extra aliasing effect, the oversampled input data will firstly be interpolation-resampled into the image positioning with a thickened GSD. Then the spatial degradation is implemented, and the degraded image will be merged into the normal GSD. On the other hand, the spectral response is simulated not by the SRF but by modeling the dispersive imaging process using the configuration parameters of the proposed Offner optical system. Besides of more coincident with the imaging process, an accompanying benefit from the proposed simulation method is that the sensor simulator can be used to validate the spectral calibration method of the proposed sensor due to the fact that the SRF of the sensor simulator is an unknown parameter and need to be calibrated. Moreover, the non-uniformity characteristics, keystone and smile effects [19], are simulated in the spatial response module and the spectral response module, respectively. The sensor would not have to be assumed as a LSI system, as the PSF used in the spatial and spectral response modules is a function of the wavelength and the location in the field of view (FOV). The rest of the paper is organized as follows: Section 2 gives a detailed description of the novel sensor simulator. Section 3 presents the simulator validation and some discussion. Section 4 provides some conclusions, and gives a perspective of the future research.

## 2. Methods

### 2.1. The Imaging Process of a Pushbroom Offner Hyperspectral Imaging Spectrometer

**Figure 1.**
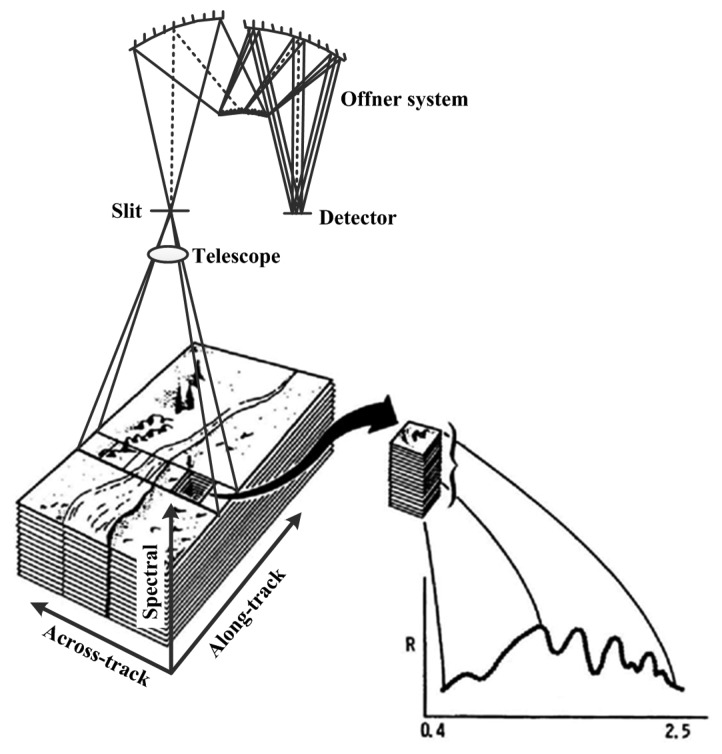
The imaging process of a pushbroom hyperspectral imaging spectrometer.

A pushbroom Offner hyperspectral imaging spectrometer is generally made up of a telescope, a slit, an Offner optical system and a detector [20]. The imaging spectrometer images a swath of a ground object, and outputs a set of dispersive images in an integration time. With the motion of the platform, a hyperspectral data cube is produced [21]. Figure 1 shows the imaging process. The telescope images a ground object swath on its focal plane, which coincides with the slit, and then the Offner system disperses the panchromatic slit image along wavelength and focuses the monochromatic images on the focal plane where the detector resides, the irradiance received by the detector is finally responded to output the DN value. Unlike the whiskbroom imaging spectrometer, the pushbroom imaging spectrometer suffers some non-uniformity effects, which are so-called the smile and keystone optical aberrations [19,22,23]. In view of the non-uniformity effects, the imaging process is accurately modeled for building the sensor simulator in this paper.

The sensor simulator is composed of a spatial response module, a spectral response module and a radiometric response module. The spatial response module simulates the imaging process of the telescope, and outputs the “panchromatic slit image”. In this module, the oversampled input data is interpolation-resampled into the correspondingly thickened detector grids and degraded by the PSFs of platform motion, jitter, telescope, slit size and detector element size. The dispersive process and the degradation of the Offner system are simulated by the spectral response module, which generates the irradiance distribution of the “monochromatic silt image” on the detector. The radiometric response module simulates the detector response and outputs the simulated DN value image.

### 2.2. Spatial Response Module

The spatial response of the hyperspectral imaging spectrometer is the imaging and degradation processes in the spatial domain. Based on the image positioning, the imaging process means resampling the oversampled input image into the corresponding detector elements. An interpolation-resampling method is developed to make a compromise between the direction error and the extra aliasing effect. The PSF, which is the function of the wavelength and the location in FOV, has the same along-track and across-track directions as the resampled image, and it is used to simulate the spatial degradation process.

#### 2.2.1. Spatial Interpolation-Resampling Method

**Figure 2.**
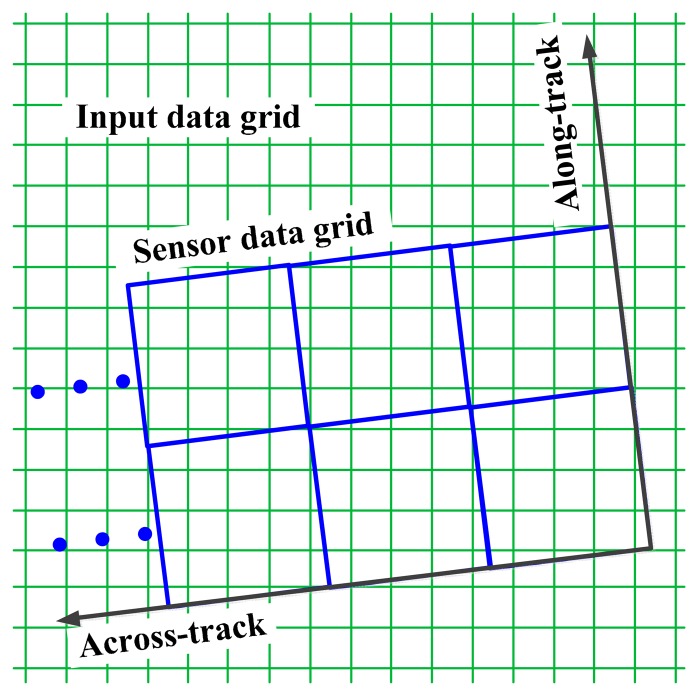
The position relationship between the oversampled input scene and the imaging data. The input scene is the little green grids which are connected by their corner points, and the bigger blue grids are the imaging pixels.

The oversampled input scene is generally georeferenced, and it is resampled into imaging data on basis of the position relationship between the input data and the imaging data, shown in Figure 2. The resampling is based on the corner coordinates of each imaging pixel, which is modeled in our previous work [24].

Figure 2 indicates that the along-track and across-track directions of sensor PSF are the same as the imaging data but different from the oversampled input data, so the convolution of the input data and the system PSF will bring some direction error.

**Figure 3.**
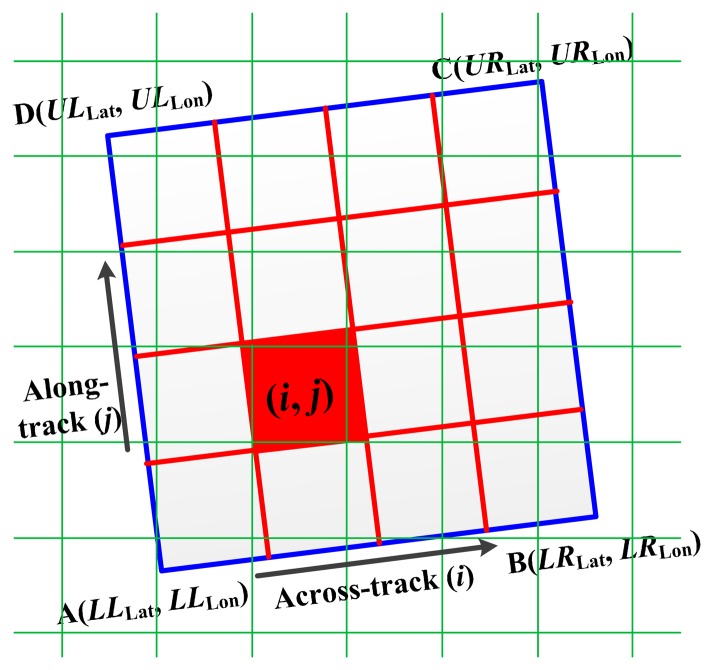
The sketch of the spatial interpolation-resampling method. In this figure, an imaging pixel is divided into 4 × 4 sub-pixels (red grid). In the interpolation-resampling process, the oversampled input data (green grid) is resampled into not the imaging pixels (blue grid) but the sub-pixels.

In order to avoid the direction error and limit the extra aliasing effect in simulated data, each imaging pixel is interpolated into *n* × *n* sub-pixels in the sensor simulator. This method is called spatial interpolation-resampling here, and the parameter *n* is used to balance the calculation quantity and the degree of the extra aliasing effect. Figure 3 shows the sketch of the interpolation-resampling process.

The lower left corner point of the imaging pixel, the point A in Figure 3, is considered as the standard point. The latitude and longitude scalars in along-track direction are noted as $\bar{AD_{\text{Lat}}}$ and $\bar{AD_{\text{Lon}}}$, and the latitude and longitude scalars in across-track direction are noted as $\bar{AB_{\text{Lat}}}$ and $\bar{AB_{\text{Lon}}}$. The four scalars are expressed by:

(1) $$\left\{ \begin{array}{l} \bar{AD_{\text{Lat}}}=\frac{1}{n}\left(UL_{\text{Lat}}-LL_{\text{Lat}}\right)\\ \bar{AD_{\text{Lon}}}=\frac{1}{n}\left(UL_{\text{Lon}}-LL_{\text{Lon}}\right)\\ \bar{AB_{\text{Lat}}}=\frac{1}{n}\left(LR_{\text{Lat}}-LL_{\text{Lat}}\right)\\ \bar{AB_{\text{Lon}}}=\frac{1}{n}\left(LR_{\text{Lon}}-LL_{\text{Lon}}\right) \end{array}\right.$$

where *UL*_Lat_ is the latitude of the upper left point of the imaging pixel, *LL*_Lat_ is the latitude of the lower left point of the imaging pixel, *LR*_Lat_ is the latitude of the lower right point of the imaging pixel, *UL*_Lon_ is the longitude of the upper left point of the imaging pixel, *LL*_Lon_ is the longitude of the lower left point of the imaging pixel, and *LR*_Lon_ is the longitude of the lower right point of the imaging pixel.

Then the corner latitude and longitude coordinates of the sub-pixel (*i*, *j*) (*i*, *j* = 1, 2, …, *n*) are expressed by:

(2) $$\left\{ \begin{array}{l} LL_{\text{Lat}}\left(i,j\right)=LL_{\text{Lat}}+\left(j-1\right)\times \bar{AD_{\text{Lat}}}+\left(i-1\right)\times \bar{AB_{\text{Lat}}}\\ LL_{\text{Lon}}\left(i,j\right)=LL_{\text{Lon}}+\left(j-1\right)\times \bar{AD_{\text{Lon}}}+\left(i-1\right)\times \bar{AB_{\text{Lon}}}\\ LR_{\text{Lat}}\left(i,j\right)=LL_{\text{Lat}}+\left(j-1\right)\times \bar{AD_{\text{Lat}}}+i\times \bar{AB_{\text{Lat}}}\\ LR_{\text{Lon}}\left(i,j\right)=LL_{\text{Lon}}+\left(j-1\right)\bar{AD_{\text{Lon}}}+i\times \bar{AB_{\text{Lon}}}\\ UR_{\text{Lat}}\left(i,j\right)=LL_{\text{Lat}}+j\times \bar{AD_{\text{Lat}}}+i\times \bar{AB_{\text{Lat}}}\\ UL_{\text{Lon}}\left(i,j\right)=LL_{\text{Lon}}+j\times \bar{AD_{\text{Lon}}}+i\times \bar{AB_{\text{Lon}}}\\ UL_{\text{Lat}}\left(i,j\right)=LL_{\text{Lat}}+j\times \bar{AD_{\text{Lat}}}+\left(i-1\right)\times \bar{AB_{\text{Lat}}}\\ LL_{\text{Lon}}\left(i,j\right)=LL_{\text{Lon}}+j\times \bar{AD_{\text{Lon}}}+\left(i-1\right)\times \bar{AB_{\text{Lon}}} \end{array}\right.$$

The radiance value of the sub-pixel is the weighted average of the input radiance locating in the sub-pixel. It is expressed by [24]:

(3) $$L_{1}=\frac{\text{∑}L_{0k}S_{k}}{\text{∑}S_{k}}$$

where *L*_1_ is the radiance of the sub-pixel, *L*_0_*_k_* is the radiance value of the input data pixel which is totally or partially locates in the sub-pixel, *S_k_* is the overlapping area between the input pixel and the sub-pixel, and ∑S*_i_* is the area of the sub-pixel.

Keystone effect refers to the inter-band spatial misregistration in imaging spectrometers. As a function of the wavelength and the location in FOV, the keystone effect of the imaging spectrometer can be described as a location offset and a scale variation of the imaging pixels in the spectral dimension [25]. In the sensor simulator, the keystone offset effect is simulated by adding an offset on the corner coordinates of the sub-pixel. The simulation of the keystone scale effect is implemented by the variation of the PSF for every imaging pixel.

#### 2.2.2. Spatial Degradation

After the interpolation-resampling process, the across-track and along-track directions of the sensor PSF are the same as the resampled image, and the spatial degradation can be implemented without suffering from the direction error. For the pushbroom Offner hyperspectral imaging spectrometer, the spatial degradation in along-track direction is due to the diffraction and aberrations of the telescope, the motion and jitter of the platform, and the slit size. In across-track direction, the image is degraded by the diffraction and aberrations of the telescope and the dispersive system, the jitter of the platform, the detector element size, and the electrons transferring and diffusing of the detector. Additionally, the degradation caused by the optical system alignment and stray light presents in both across-track direction and along-track direction. The radiance of the sub-pixels in the sensor simulator is not the interpolation of the input image but the weighted average, and the sub-pixels will be merged after the spectral response module. Thus, the degradation effects of the slit size and the detector element size are contained in the resampling and mergence processes, and they are not implemented in the spatial degradation repeatedly. Moreover, the Offner system disperses the slit image and focuses the monochromatic images onto the focal plane. As a result, the Offner system degrades the imaging data in the across-track direction of the spatial dimension and the spectral dimension synchronously, which is the plane expanded by the across-track axis and the spectral axis in Figure 1. Therefore, the degradation of the Offner system will not be implemented here but in the spectral response module. PSF is the matrix multiplication of the LSFs, and the LSFs used in the sensor simulator are presented following.

MTF is widely used to evaluate the spatial performance of an imaging system [26], and the MTF values of the telescope and the dispersive system can be obtained by the commercial optical design packages, such as Zemax and Code V. Thus, with the assumption that the LSF of the optical system is Gaussian distributed, the LSF can be deduced from the MTF at the Nyquist frequency (*MTF*_Nyq_). In this work, the LSF of an optical system is expressed by:

(4) $$\text{LSF}\left(x\right)=\exp\left(-\frac{x^{2}}{2\sigma^{2}}\right)$$

where $\sigma =\frac{\sqrt{2}}{\pi }\sqrt{\text{In}\left(\frac{1}{MTF_{\text{Nyq}}}\right)}$ is the standard deviation.

Motion blur occurs in the aero and orbital images, and the LSF, which is caused by the linear motion of the platform, is expressed by a rectangular function [27]:

(5) $$\text{LSF}\left(x\right)=\text{rec}\left(\frac{x}{v\times t}\right)$$

where *v* is the platform velocity, and *t* is the integration time of the imaging spectrometer. The jitter LSF comes from the high frequency line of sight vibrations, compared to the integration time. For the random and isotropic vibrations with a standard deviation of σ_jitter_ (pixel), the jitter LSF is expressed by [27]:

(6) $$\text{LSF}\left(x\right)=\frac{1}{\sqrt{2\pi }\sigma_{\text{jitter}}}\exp\left(-\frac{x^{2}}{2\sigma_{\text{jitter}}^{2}}\right)$$

Generally, the degradation of the electrons transferring and diffusing of the detector is extremely slight, so it is ignored in the sensor simulator. The LSFs in the same direction are combined by the convolution. Then the PSF of the system is obtained by the matrix multiplication of the along-track LSF and the across-track LSF. The MTF of the optical system used in the sensor simulator is a function of the wavelength and the location in FOV, thus, the PSF is not constant as well. Unlike the traditional sensor simulator, the sensor would not have to be assumed as a LSI system in the work.

### 2.3. Spectral Response Module

The Offner optical system is the core part of the imaging spectrometer. The standard concentric Offner system is made up of a big concave mirror and a small convex mirror. Chrisp made a significant improvement on it, and he replaced the big concave mirror with two smaller concave mirrors, which receives the slit image and the dispersive images respectively [28]. The improved configuration has a compact size and flat meridional and sagittal focal planes. Based on the Rowland circle equipment, Prieto-Blanco further studied the meridional and sagittal focusing curves and provided a method to design an Offner imaging spectrometer [29].

**Figure 4.**
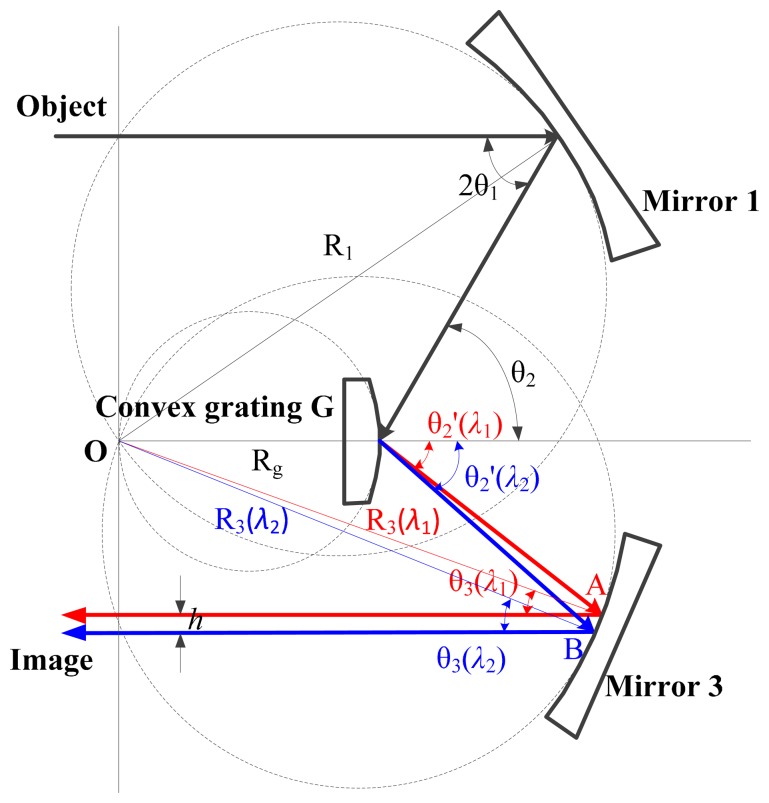
An Offner imaging spectrometer with Rowland circle condition.

Figure 4 shows an Offner imaging spectrometer with Rowland circle condition. The centers of the object plane, the image plane and the concentric system lie in a line. The central ray of the cone of light, called as the main light here, is used to analyze the light path. Then Rowland circle condition satisfies the following equations [29,30]:

(7) $$\left\{ \begin{array}{l} R_{\text{g}}\sin{\theta_{2}}^{\prime }\left(\lambda_{1}\right)=R_{3}\sin\theta_{3}\left(\lambda_{1}\right)\\ R_{\text{g}}\sin{\theta_{2}}^{\prime }\left(\lambda_{2}\right)=R_{3}\sin\theta_{3}\left(\lambda_{2}\right) \end{array}\right.$$

where *R*_g_ is the radius of the convex grating, θ_2_ ′ is the diffraction angle of the grating, *R*_3_ is the radius of the Mirror 3 in Figure 4, θ_3_ is the exit angle of the Mirror 3, and λ_1_ and λ_2_ represent the wavelength.

In Figure 4, the diffraction lights of wavelength λ_1_ and λ_2_ project at the points A and B of Mirror 3 respectively. Points A and B are both close to the tangent point of Mirror 3 and its Rowland circle. Because of $R_{3}\gg \hat{AB}$, it is reasonable to consider that *R*_3_(λ_1_) ≈ *R*_3_(λ_2_) ≈ *R*_3_, where *R*_3_(λ_1_) and *R*_3_(λ_2_) are the distance of OA and OB in Figure 4 respectively. The emergent lights from points A and B project on the focal plane. The distance of the projections *h* is expressed by:

(8) $$h=R_{3}\left(\lambda_{2}\right)\sin\theta_{3}\left(\lambda_{2}\right)-R_{3}\left(\lambda_{1}\right)\sin\theta_{3}\left(\lambda_{1}\right)\approx R_{3}\left(\sin\theta_{3}\left(\lambda_{2}\right)-\sin\theta_{3}\left(\lambda_{1}\right)\right)$$

where θ_3_(λ_1_) and θ_3_(λ_2_) are the exit angles of the lights with wavelength λ_1_ and λ_2_ in Figure 4, respectively.

Substituting Equation (7) into Equation (8), the distance of the monochromatic slit images with different wavelength *h* is obtained:

(9) h=Rg(sinθ2'(λ2)−sinθ2'(λ1))

The grooves spacing of a Rowland ruled convex grating is not constant around the grating surface, but it is constant along the chord. Moreover, the facet angle of the grating remains constant with respect to that chord. As a result, the incidence angles of a convex grating for the whole FOV are approximately constant. The exit angle for the diffraction light with wavelength λ can be obtained by the grating equation, which is expressed as:

(10) $$d(\sin\theta_{\text{exit}}-\sin\theta_{\text{in}})=m\lambda$$

where *d* is the grating period, θ_exit_ represents the exit angle, θ_in_ represents the incidence angle, *m* is grating operating order, and λ represents the proposed wavelength.

With the configuration parameters of the Offner imaging spectrometer presented, Equations (9) and (10) indicate the projections on the detector for every wavelength. Although the spectral resolution of the input data is very high, every band has a bandwidth. The SRF of the input data is supposed to be rectangular, as a result, the wavelength range for every band is [λ*_i_* − 0.5 × *bw_i_*, λ*_i_* + 0.5 × *bw_i_*], where λ*_i_* is the central wavelength of band *i*, and *bw_i_* is the band width of band *i*. Considering Equation (10), the diffraction angles of the boundaries of band *i* are:

(11) $$\left\{ \begin{array}{l} \sin\theta_{\text{d}i+}=\frac{m\left(\lambda_{i}+0.5\times wb_{i}\right)}{d}+\sin\theta_{\text{in}}\\ \sin\theta_{\text{d}i-}=\frac{m\left(\lambda_{i}-0.5\times wb_{i}\right)}{d}+\sin\theta_{\text{in}} \end{array}\right.$$

Relative to the reference wavelength, the projections of the boundaries at the detector are:

(12) $$\left\{ \begin{array}{l} s_{i+}=\left(\sin\theta_{\text{dc}}-\sin\theta_{\text{d}i+}\right)\times R_{\text{g}}\\ s_{i-}=\left(\sin\theta_{\text{dc}}-\sin\theta_{\text{d}i-}\right)\times R_{\text{g}} \end{array}\right.$$

where θ_dc_ is the diffraction angle of the reference wavelength. Then the monochromatic slit image of band *i* covers the range of [*s_i_*_−_, *s_i_*_+_] on the detector. The smile property is a center wavelength shift in across-track direction [31]. It is simulated by adding a location shift on the *s_i_*_−_ and *s_i_*_+_. The shift is the function of the wavelength and the location in FOV.

After the calculation of the boundary locations for each band of the spectrally oversampled input data, the irradiance received by each detector element is obtained. As in the aforementioned analysis, the Offner system degrades the imaging data in across-track direction and the spectral dimension synchronously. For the degradation operation, the spacing in spectral dimension needs to be the same as the spatial dimension. Thus a detector element is divided into *n* × *n* sub-elements monospaced. Like the spatial degradation, the MTF of the Offner system is used to calculate the PSF for every sub-element, and the degradation is implemented by convolving the PSF with the irradiance image received by the detector. After the degradation, the irradiance image is assembled (merging *n* × *n* sub-elements into a pixel).

### 2.4. Radiometric Response Module

**Figure 5.**
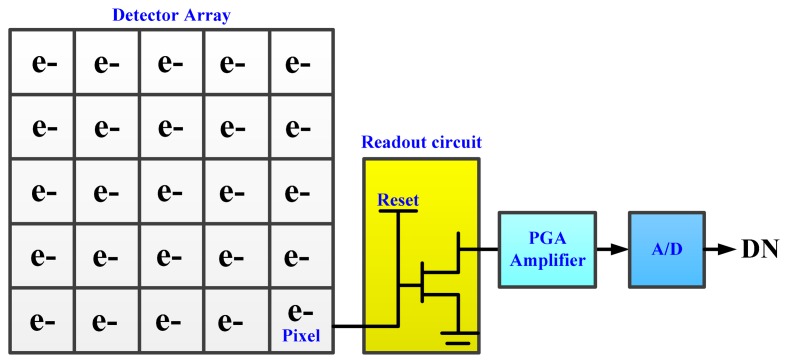
The sketch of detector and electronics system.

The detector received irradiance is responded to stimulated electrons, then the electrons are transferred, amplified and A/D transformed into the output DN value. The transfer process, which is shown in Figure 5, is simulated in the radiometric response module.

The first step is the calculation of the number of stimulated electrons (signal electrons) freeing from a detector element by equation [32]:

(13) $$n_{0}=\frac{\pi }{4}{\left(\frac{D}{f}\right)}^{2}A_{\text{d}}t\overset{\lambda_{2}}{\underset{\lambda_{1}}{\text{∑}}}\tau \left(\lambda_{i}\right)\eta_{\text{d}}\left(\lambda_{i}\right)L\left(\lambda_{i}\right)\frac{\eta_{\text{e}}\left(\lambda_{i}\right)}{h\upsilon_{i}}\eta \left(\lambda_{i}\right)bw_{i}$$

where *D* is the pupil aperture, *f* is the focus, *A*_d_ is the area of the detector element, *t* is the integration time, τ is the transfer efficiency of the telescope, η_d_ is diffraction efficiency of the Offner system, *L* is the input at-sensor radiance, η_e_ is the quantum efficiency of the detector element, *h* is the Planck's constant, *ν_i_* is the frequency of the wavelength λ*_i_*, η is the ratio of the wavelength range of band *i* which lies on the element to the band width, and λ_1_ and λ_2_ are the wavelength boundaries which will locate in the element.

The DN values fluctuate due to the influence of noise. The considered noises are the photon noise, the dark noise, and the readout noise. The noise sources are the fluctuation of the signal electrons (photon noise), the dark current electrons (dark noise), and the readout circuit (readout noise, which comprises the KTC noise, amplifier white noise, 1/*f* noise, and so on), respectively. The photon noise and the dark noise follow the Poisson distribution, which can be described as the Gaussian distribution for a large number of samples. Here, the ggreadout noise is also assumed to be Gaussian distributed for simplifying the calculation. Thus the standard deviation of the total noise is expressed as:

(14) $$\sigma_{\text{noise}}=\sqrt{n_{0}+n_{\text{d}}\times t+n_{\text{readout}}\times n_{\text{readout}}}$$

where *n*_d_ is the dark electrons that a detector element generates in one second and *n*_readout_ is the readout noise. The total electrons are the summation of the signal electrons, dark current electrons, and the noise. Considering the process of the amplifying and the ADC, the DN value is expressed as:

(15) $$DN_{0}=\left(2^{b}-1\right)\frac{n_{\text{total}}R_{\text{c}}}{V_{\text{ref}}}$$

where *b* is the radiometric resolution, *n*_total_ is the total electrons, *R*_c_ is the conversion gain, and *V*_ref_ is the reference voltage. The non-uniform effect (including the striping artifacts and dead pixels in some literatures) is simulated by a gain parameter on the *DN*_0_, and the nonlinearity effects can be simulated by a look-up-table (LUT) [13].

## 3. Validation and Discussion

In this section, the spatial, spectral, and radiometric calibration processes of an imaging spectrometer in the laboratory are simulated, and the calibration results (MTF, SRF, and radiometric calibration parameters) are used to design experiments to validate the spatial, spectral and radiometric responses of the sensor simulator.

### 3.1. Spatial Response Validation

The spatial characteristics of an imaging system can be expressed by the MTF parameter, which is widely used in the spatial response evaluation of IKONOS, Quickbird, Hyperion, and so on [5,33]. In this work, the edge method [33] is implemented to calculate the MTF of the sensor simulator. A synthetic edge scene is “imaged” by the traditional sensor simulator and the proposed sensor simulator in the validation experiment. The MTFs from the two simulated data are compared with the theoretical MTF to validate the spatial response of the proposed sensor simulator in the paper.

**Figure 6.**
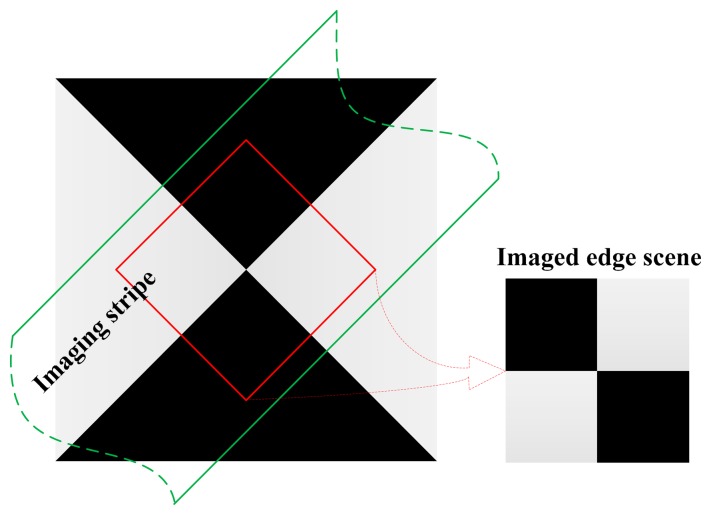
The synthetic edge scene. The left is the synthetic edge scene, the region enclosed by green line is the imaging stripe, and the right is the imaged edge scene.

The first step is to construct an edge scene. In this work, an oversampled synthetic edge scene with the edge azimuth of 45° is built, and the platform also moves along the azimuth of 45°. Figure 6 shows the edge scene and the imaging stripe. The horizontal edge and the vertical edge of the imaged scene (the right in Figure 6) are used to calculate the along-track MTF and across-track MTF, respectively. In order to simplify the comparison, the MTF values (at the Nyquist frequency) of the telescope and the Offner system in along-track direction and in across-track direction are all assumed to be constantly 0.8. It is also supposed that the alignment and stray light MTF is a constant 0.8, the platform moves a pixel during an integration time, and the standard deviation of the jitter is 0.1 pixels. The MTFs of the jitter and the motion are the Fourier transform of the LSFs. Moreover, the slit size, the detector element size, and the motion have the same MTF. Theoretically, the MTFs of the sensor in along-track and across-track directions at the Nyquist frequency are:

(16) $$\left\{ \begin{array}{l} MTF_{\text{AL}}=MTF_{\text{telescopeAL}}\times MTF_{\text{motion}}\times MTF_{\text{jitter}}\times MTF_{\text{slit}}\times MTF_{\text{alignment}}=0.2469\\ MTF_{\text{AC}}=MTF_{\text{telescopeAC}}\times MTF_{\text{OffnerAC}}\times MTF_{\text{jitter}}\times MTF_{\text{detector}}\times MTF_{\text{alignment}}=0.3103 \end{array}\right.$$

where *MTF*_telescope_, *MTF*_Offner_, *MTF*_motion_, *MTF*_jitter_, *MTF*_slit_, *MTF*_detector_, *MTF*_alignment_ are the MTF caused by the telescope, Offner system, platform motion, platform jitter, slit size, detector element size, alignment and stray light, respectively; and the subscripts *AL* and *AC* represent the along-track direction and the across-track direction, respectively. Generally, the MTF in along-track direction is smaller than in across-track direction because of the platform motion [14].

**Figure 7.**
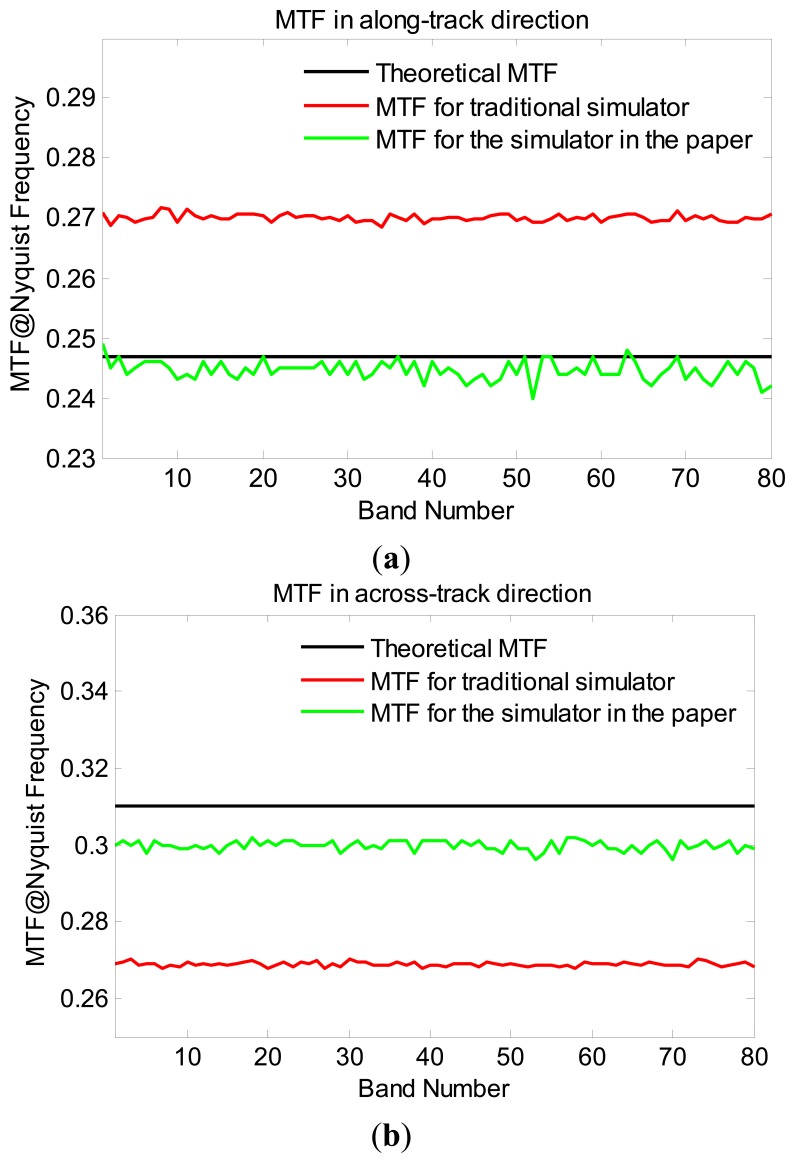
The MTF (Nyquist frequency) calculated from the simulated edge scenes for the traditional simulator and the proposed simulator. (**a**) MTF in along-track direction; (**b**) MTF in across-track direction.

The synthetic scene is “imaged” by the traditional sensor simulator and the proposed sensor simulator along the green stripe, respectively. The along-track and across-track MTFs calculated from the imaged edge scenes are shown in Figure 7. Compared with the MTF results derived from the traditional sensor simulator imaged data, the MTF results of the proposed sensor simulator is much closer to the theoretical results. The fluctuations of the calculated MTF curves are due to the noise in the simulated data, which affects the stability of the edge method. The mean errors of the MTF for the traditional sensor simulator are 9.36% and 13.31% in along-track direction and across-track direction, respectively. As previously mentioned, the serious MTF error comes from the direction error. For the proposed sensor simulator, the mean errors are 0.96% and 3.37% in the along-track direction and in the across-track direction, respectively. Obviously, the proposed sensor simulator avoids the direction error effectively and advances the simulation accuracy of the imaging blurring greatly. It should be noticed that the sampling interval ratio between the input data and the simulated data results in some effects on the accuracy of the simulations [34], and the ratio used in this work is 7.0.

**Figure 8.**
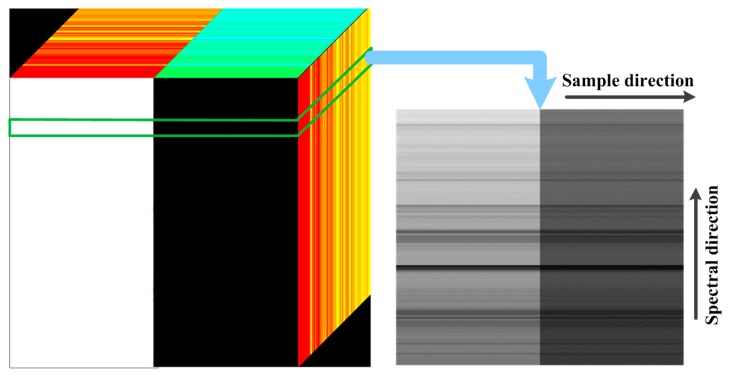
The imaging data. The left is a data cube, and the right is the spectral image for a line, which is not a standard edge image.

While Figure 7 also shows that the MTF error in across-track direction is slightly larger than that in along-track direction for the proposed sensor simulator. This phenomenon is caused by the spatial-spectral degradation in the spectral response module, as the spectral image of an imaging line is not a standard edge image but a stripe image. A spectral image is shown in Figure 8. Therefore, the data from different wavelength (different line in the right figure of the Figure 8) interplays when the spatial-spectral degradation process is implemented. The interplay brings an extra blurring effect which results in a slight MTF falling.

### 3.2. Spectral Response Validation

Generally, the SRF, which is produced by the spectral calibration, is used to evaluate the spectral response for the multispectral and hyperspectral imaging spectrometers [35,36]. Unlike the general sensor simulator, the output DN value in this work does not have a known SRF, so the SRF of the sensor simulator need to be calibrated. The spectral calibration methods include the monochromator method and the absorption feature method (emission lamp or atmosphere absorption feature) [37]. The monochromator method is used in the laboratory spectral calibration with all the bands’ SRF produced, while the absorption feature method is commonly used in the in-flight calibration applying partial bands’ SRF. Therefore, the monochromator method is employed to calibrate the sensor simulator in this work.

**Figure 9.**
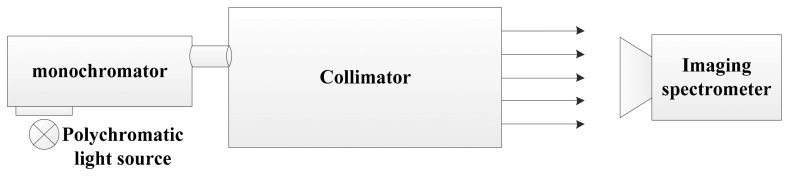
The sketch of the monochromator method for the spectral calibration.

Figure 9 shows the configuration sketch of the monochromator method [38]. The polychromatic light illuminates the slit of the monochromator, and the output monochromatic light is expanded to fulfill the FOV of the imaging spectrometer. The center wavelength and the full width half maximum (FWHM) of the monochromatic light are changed by varying the grating angle and the slit size of the monochromator. Simulating this process, a uniform monochromatic light image is produced firstly. Then the monochromatic image is “imaged” by the sensor simulator, and outputs the DN value. The same operation is repeated with the wavelength of the monochromatic image stepwise upping. For the grating hyperspectral imaging spectrometer, Gaussian function is widely used to describe the SRF shape [5,35]. Hence, these simulated DN value images are finally fitted by the Gaussian function. Here, the Gaussian function is defined as following equation:

(17) $$g\left(\lambda \right)=k_{0}\exp\left(\frac{{\left(\lambda -\lambda_{0}\right)}^{2}}{2{\sigma_{g}}^{2}}\right)+b_{0}$$

where *k*_0_ is a gain parameter, *b*_0_ is a bias parameter, λ_0_ is the center wavelength of the fitted spectral band, σ_g_ is the standard deviation, and the band width of the fitted spectral band is $F\text{WHM}=2\sigma \sqrt{2\text{ln}2}$. The smile parameter is presented by implementing the same fitted operation to the whole FOV.

**Figure 10.**
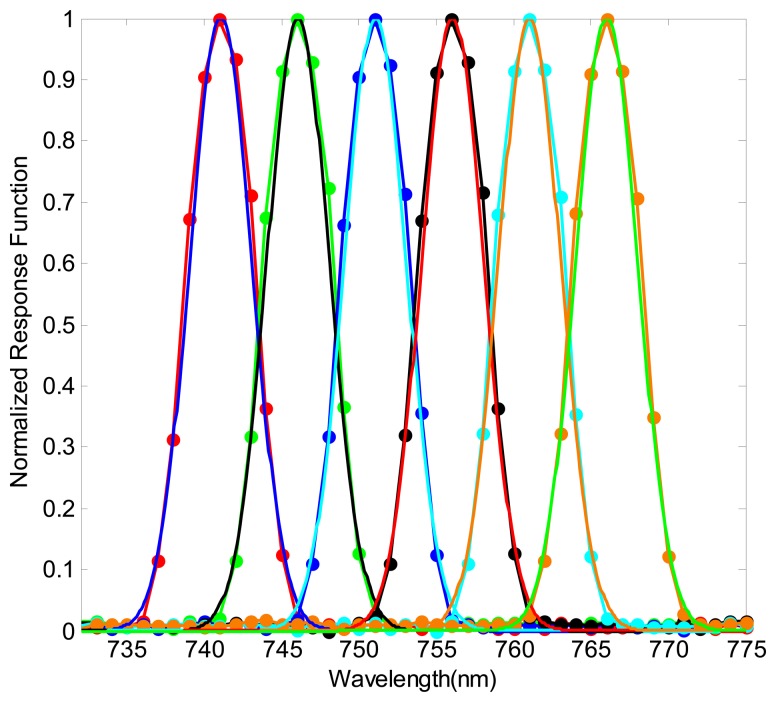
The SRFs obtained with a simulated scanning monochromator. The curves with spots are the normalized simulated DN value, and the other curves are the fitted results.

A VNIR hyperspectral imaging spectrometer [39], whose spectral resolution is about 5 nm, is simulated to validate the spectral response of the sensor simulator. Some calibration results of the sensor simulator are shown in Figure 10. It can be seen that Gaussian function accords with the SRF of the sensor simulator greatly. Several SRFs (locating at the center of FOV) of the imaging spectrometer are calibrated, and the comparison of the simulated results and true results is detailed in Table 1. It can be seen that the SRF of the sensor simulator is very close to the true result in bands 11 and 31, and the errors of the center wavelength and the FWHM are 2.57% and 0.86%, respectively. However, the errors in band 41 are 43.06% and 2.07%. The spectral resolution of a grating imaging spectrometer is constant in theory. Therefore, the deviation is most probably caused by the manufacture error of the imaging spectrometer which leads to some difference between the input parameters and the true configuration parameters.

Moreover, the sensor simulator can also be used to validate the spectral calibration method by applying tradeoff studies to characterize the impact of the monochromator parameter choices, such as the stability and the FWHM.

### 3.3. Radiometric Response Validation

**Figure 11.**
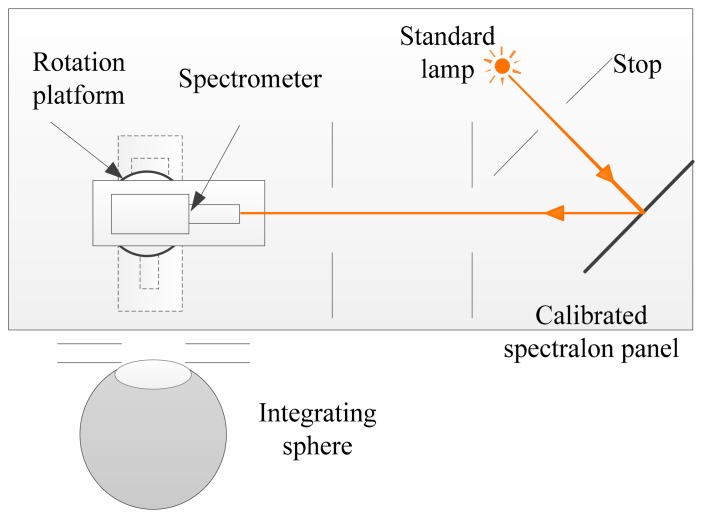
The configuration of the standard-lamp-based radiometric calibration in the laboratory.

The radiometric response of the sensor simulator is evaluated by the radiometric calibration. The laboratory radiometric calibration (standard-lamp-based method) is simulated to calibrate the sensor simulator. The calibration configuration is shown in Figure 11 [40,41]. First, the spectrometer, which is made up of a monochromator and a photomultiplier tube, is calibrated by a standard lamp and a spectralon panel. Then tuning the rotation platform, the integrating sphere is calibrated by the spectrometer. Finally the calibrated integrating sphere is used to calibrate the proposed imaging spectrometer. Every measurement process will have an uncertainty, so the above stated measurements also have uncertainties. When the analysis of the calibration uncertainty effects on the quality of the restored radiance data is demanded, these uncertainty caused by the measurement process should be considered.

#### 3.3.1. Radiometric Calibration Uncertainty

Using the calibrated integrating sphere as the light source, the at-sensor radiance of the imaging spectrometer is expressed as:

(18) $$L=\frac{V'\times \rho \times E}{V\times \pi }$$

where *V* ′ is the response of the spectrometer when the integrating sphere is the light source, ρ is the reflectance of the spectralon panel, and *E* and *V* are the irradiance spectralon panel received and the response of the spectrometer when the standard lamp is the light source.

Each measurement of the parameters in Equation (18) has an uncertainty. Generally, the total uncertainty of the measured parameters, which equals to the root sum square of the partial uncertainty, is considered as the radiometric calibration uncertainty [42]. What is more, each measured parameters has its uncertainty distribution, which follows the Gaussian function on basis of the central limit theory. Therefore, the uncertainty distribution of the *L* is the synthesis of the partial uncertainty distribution, which is expressed as [41]:

(19) $$f\left(L\right)=\frac{1}{\sqrt{2\pi }\sigma_{\text{c}}}e^{-\frac{{\left(L-L_{0}\right)}^{2}}{2\sigma_{\text{c}}^{2}}}$$

where *L*_0_ is the truth value of the *L* and σ_c_ is the synthesized uncertainty. The radiometric calibration uncertainty is simulated by adding the synthesized uncertainty on the radiance of the integrating sphere.

#### 3.3.2. Radiometric Calibration of the Sensor Simulator

The detector generally works in the linear response region, thus there is a linear relationship between the simulated DN value and the at-sensor radiance [43]:

(20) L=A×DN+B

where *A* and *B* are the gain and bias matrix of the calibration parameters, respectively. Considering the calibration uncertainty of 5%, a random error, which is distributed as Equation (19), is incorporated into the at-sensor radiance during the calibration process. Imaging two uniform scenes from the calibrated integrating sphere 15 times and averaging them, respectively, the calibration matrix parameters are obtained with the dark current subtracted. If the detector suffers from the response nonlinearity, the nonlinearity should be calibrated and corrected before the radiometric calibration [13].

**Figure 12.**
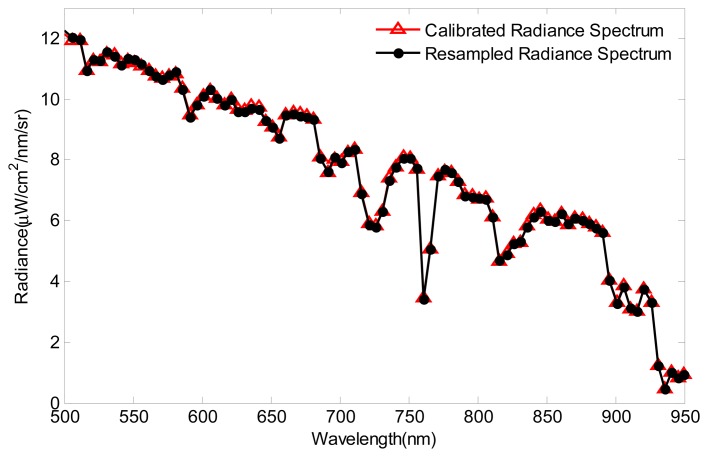
The comparison of the calibrated radiance spectrum and the spectrally resampled spectrum.

For avoiding the spatial degradation effect in the simulating process, a uniform radiance scene, composed of the MODTRAN-simulated radiance spectrum [44], is built as the test data in the radiometric response validation experiment. The MODTRAN parameters are Mid-Latitude Summer, Rural aerosol model with 23 km visibility, elevation angle 30°, and ground albedo 0.5 constant. The uniform scene is “imaged” by the sensor simulator and calibrated to output the restored radiance data. In order to validate the radiometric response of the sensor simulator, the calibrated spectrum is compared with the standard spectrum, which is obtained by the spectral resampling of the uniform scene. The SRF, used for the spectral resampling, is the spectral calibration result of the sensor simulator. The comparison of the calibrated radiance spectrum and the spectrally resampled spectrum is shown in Figure 12. It can be seen that the two spectra are almost overlapped, and the slight deviation is caused by the sensor noise and the calibration uncertainty. The comparison indicates that the radiometric response simulation of the sensor simulator and the calibration simulation process are both valid.

## 4. Conclusions

In this paper, a novel sensor simulator for the pushbroom Offner hyperspectral imaging spectrometer has been presented. The simulator is comprised of a modular structure and includes the entire image data acquisition chain of the sensor. The simulator with the input of the spatially and spectrally oversampled scene data outputs the simulated DN value data with the proposed imaging spectrometer characteristics. The non-uniformity effects of the sensor, such as keystone and smile, can be simulated in the corresponding modules. The calibration processes were implemented to validate the sensor simulator through the respect of spatial response, spectral response and radiometric response, respectively. The validation experiments proved that the modules have highly accurate performance. Thus, the developed sensor simulator enables a detailed analysis of the effects of different instrumental configuration parameters on the imaging process, and will contribute to a great convenience to the instrument design.

Although the parameters and the processing steps are optimized for the pushbroom Offner hyperspectral imaging spectrometer, the sensor simulator is implemented in a modular and flexible way so that different optical sensors can be simulated by adjusting the spectral response module. Future work will focus on the preprocessing method development based on the simulated data. The uncertainty factors can be considered in the spectral and radiometric calibrations, thus the effect of these factors on the data application also need be further analyzed.

## Figures and Tables

**Table 1.** The SRF comparison of the simulated calibration results of the sensor simulator and the results of the true sensor.

**Band Number**	**The True SRFs**		**The Simulated SRFs**	

	**Center Wavelength**	**FWHM**	**Center Wavelength**	**FWHM**
11	551.19 nm	4.67 nm	551.31 nm	4.71 nm
31	651.34 nm	4.67 nm	651.19 nm	4.71 nm
41	699.05 nm	4.83 nm	701.13 nm	4.73 nm
